# From cancer to rejuvenation: incomplete regeneration as the missing link (Part I: the same origin, different outcomes)

**DOI:** 10.2144/fsoa-2019-0119

**Published:** 2020-01-14

**Authors:** Mamuka G Baramiya, Eugene Baranov

**Affiliations:** 1 AntiCancer, Inc., San Diego, CA 92111, USA; 2 Stemedica Cell Technologies, CA 92121, USA

**Keywords:** aging, carcinogenesis, epigenetic, immunological tolerance, immunoprivileged sites, morphostasis, regeneration, rejuvenation, reontogenesis, senescence

## Abstract

Here, we interpret malignant tissue transformation from the aging point of view, that is, as a result of insufficient cell adaptation to the needs of regeneration/repair and proliferation. A consequence of the aging (senescence) process is gradual loss of self-renewal potential. It limits lifespan and leads to death due to the decline of tissue/organ functions, failure of regulatory mechanisms, disruption of endogenous processes and increased susceptibility to exogenous factors. Recapitulation of the embryonic pathway of self-renewal/rejuvenation in adulthood is epigenetically determined. At the postembryonic stage, in the absence of immune privilege, this recapitulation is transformed into cancer (potency expansion of single structures composing the organism to the detriment of the whole organism or disintegrating growth). We suggest that the process of rebirth occurs in the same way as embryonic tissue growth. Thus, the idea to use the potential of the transformed cells to stop the aging process has been proposed.

## Lay abstract

This article interprets malignant tissue transformation from the aging point of view, or in other words, as a result of insufficient cell adaptation to the needs of repair and proliferation. We suggest that the process of rebirth, or complete quantitative and qualitative restoration of tissues resulting in reversal of the aging process, occurs in the same way as embryonic tissue growth. The idea to use the potential of the transformed cells to stop the aging process is proposed.

*Someday perhaps it will turn out to be one of the ironies of nature that cancer, responsible for so many deaths, should be so indissolubly connected with life.* Charles Oberling [1]

According to previously formulated concepts [2,3], the derepression of the embryogenesis program develops as an attempt of living matter to restart/recapitulate the morphogenetic modules of early ontogenesis (embryogenesis). This is in order to implement the processes of effective, full and unlimited self-renewal where and when the current development program of the organ/tissue is critically insufficient for proper functioning. In nature, the ability to restore body parts and even the whole organism is always associated with tissue respecification, that is, recapitulation of the morphogenetic module (blastema), which is possible only under recapitulation of the embryonic developmental path. In pregnancy, it is the immune privilege of the fetus that ensures the unidirectionality of the vector totipotency to differentiation, or integrating growth (IG). IG is defined here as the submission of potency of single cells composing an organism to the development program and functions of the whole organism. However, in the adult organism, in the absence of immune privilege, this recapitulation is transformed into cancer, or disintegrating growth (DG). DG is defined here as a priority of extension potency of single cells over the development program and functions of the whole organism. The reason for this is that in conditions of tissue/function deficiency, the immune system exhibits its inherent morphogenetic potential for maintaining cellular/tissue homeostasis (morphostasis), blocking the potential for cell redifferentiation, which, as it turns in reverse, leads to uncontrolled deployment of the development program in the opposite direction. That is, where the morphogenetic module is susceptible to embryonic induction, we have morphogenesis, and where not, we have carcinogenesis.

One of the main (noninfection-related) functions of the immune surveillance system is to maintain cellular (tissue) homeostasis (morphostasis) that is carried out through maintaining the growth processes in damaged tissues. In this process, the immune response to proliferating cells is carried out through the mechanism of active tolerance, when there is an immune response; however, it does not kill, but supports. The standard regenerative module during regeneration works within the program of relatively rigid-specific determination, and the level of tissue-specific differentiation is determined by the principle of submitting the interests of single units to the interests of the entire organism (IG). The active tolerance reaction ensures stability of this particular program. In the case of reontogenesis, that is, the recapitulation of morphogenetic modules of early ontogenesis (embryonic development program) with a specific tendency to autonomy, the same interactions ensure the stability of this particular inverse vector (expanding the potential of individual units that is detrimental to the entire organism, that is, DG or cancer). In other words, the immune response ‘bluntly’ supports a growth program, which is launched when an absolutely critical or relative deficiency of tissue/functions occurs; regardless of whether the regenerative (within differentiation) or embryonic (with dedifferentiation and progressive autonomy) module works currently. The same takes place during pregnancy at the mother–fetus interface. However, during pregnancy, it happens at the interface of multinucleated, nondividing syncytium (syncytiotrophoblast), and the embryo is protected in the immunoprivileged zone. Due to this protection, the embryo gradually advances from totipotency to differentiation (the same IG).

Therefore, DG is only epigenetically blocked differentiation, incomplete somatic embryogenesis or fragmented/unimplemented morphogenesis. The development of full immunological tolerance to the markers of the embryonic pathway (the so-called tumor-associated antigens) will allow avoidance of DG. Then, the embryonic pathway recapitulation, starting where and when aging/involution within the definitive structures has acquired an irreversible character, will be carried out as a closed cycle (rejuvenation circle) of dedifferentiation, redifferentiation, dedifferentiation, redifferentiation (rejuvenation, senescence, rejuvenation, senescence and so on. At the cellular level, this will eliminate senescence, as the final stage of the fate of cells, making it only a transitional stage. At the level of the whole organism, this will allow achievement of permanent full quantitative and qualitative self-renewal/rejuvenation and elimination of aging. We are ‘pregnant’ with life, and we need not necessarily think about futile attempts to killing the cancer, which with conventional treatment approaches can return, but instead look to a qualified obstetrician for competent obstetric care.

## Cancer is an unfinished attempt of rejuvenation

The methylation landscape in cancer is heterogeneous. A large part of the genome is hypomethylated, while a small part of the genome is hypermethylated [4]. The methylation/demethylation DNA pattern is strikingly similar in many types of tumors [5], indicating the universality of this process in carcinogenesis in all types of cancer. An entire genome demethylation in tumors (also a specific feature of the embryonic type of self-renewal) is often accompanied by hypermethylation of some parts (∼20%) of the CpG islands of regulatory sites, which are nonmethylated in mature somatic differentiated cells. This is a characteristic feature of the main mechanism of inactivation of the so-called tumor-suppressor genes [6–8]. It is known that overall DNA hypomethylation, especially in intragenic regions, is a common feature of most tumors. At the same time, it has been shown that an increase in DNA methyltransferase activity precedes malignant transformation in cell cultures [6]. This is in line with data that show that cells accumulate aberrant methylation as a feature of aging, and therefore aberrant methylation, as a function of aging, precedes the appearance of tumors [9,10]. Furthermore, DNA hypomethylation in cancer cells is primarily linked to the repressive H3K9me3mark, which is known to block the reprogramming of somatic cells into induced pluripotent stem cells by preventing the incorporation of reprogramming pluripotency factors (e.g., Oct4, Sox2, Klf4, c-myc) onto target genes [11]. A concept shared by some researchers that aging saves us from cancer (through a number of mechanisms, such as cell cycle arrest, the activity of the ‘guardian of the genome’ [*p53*], the lack of TERT activity and others) appears logical and speculatively understandable. However, this is only true until the increasing shortage of tissues and functions and the aging senescence-associated secretory phenotype (SASP) signaling, through positive feedback, fail to eliminate these deficiencies through the recapitulation of morphogenetic modules of the early stages of ontogenesis (embryonic development), with its high self-renewal potential characteristic. It should be noted that the hypomethylation of growth-proliferative genes through demethylation caused by methyltransferase inhibition leads to the elimination of SASP through an increase of antisenescence *TERT, bFGF, VEGF* and *ANG* gene expression and a decrease of senescence-related *ATM*, *p21* and *p53* gene expression [12], which facilitates carcinogenesis and supports cancer cell growth and survival via the CXCL12/CXCR4 axis [13], which is also involved in the regulation of maternal natural killer (NK) cells by fetal trophoblast, resulting in the transformation of blood NK cells into decidual NK cells (NK regs) at the fetal–maternal interface with acquisition of immune tolerance characteristics for successful pregnancy [14]. This is important for understanding the commonality of the underlying mechanisms of cancer, permanent self-renewal and early stages of ontogenesis.

Cyclin-dependent kinase inhibitor and a main regulator of the G1/S transition checkpoint *P21* are important transcriptional targets of *p53*. The activation of *P21* results in cell cycle arrest, and in case of prolonged damage, it leads to cellular senescence. Activation of PUMA, BAX and BAK induces apoptosis. Their activation also leads to tissue degeneration and loss of function as well as depletion of stem cells and impaired renewal potential. All of these are hallmarks of the aging process [15]. Studies using different mouse models with *p53* hyperactivation demonstrated an increased resistance of mice to carcinogenesis, but premature aging and lifespan shortening [16–18]. However, neither type of mouse model showed cancer or aging elimination. On the other hand, the lack of the essential effectors of *p53*-induced apoptosis in gene-targeted mice did not lead to spontaneous tumor occurrence [19].

Self-renewal capacity, such as the Wnt signaling pathway, is often deregulated in aged organisms. The accumulation of DNA damage and the activation of tumor-suppressor signaling pathways are other important factors underlying the reduced ability of the stem cells to regenerate and repair damaged tissues. There are two main tumor-suppressor signaling pathways, p53/p21 and p16INK4a/pRB, which result in cell growth arrest and, in the case of persistent stimuli, lead to senescence. Hypomorphic BubR1 mice show premature separation of sister chromatids, leading to progressive aneuploidy. They developed a characteristic progeroid phenotype with high p16INK4a expression in skeletal muscle and fat tissue. The suppression of p16INK4a in these mice resulted in increased longevity, a delay in their typically degenerative phenotype and a reduction of the accumulation of senescent cells. All these evidences support p16INK4a involvement in senescence and age-related pathologies [20]. Staining for Ki67, p16 and SA-β-Gal has shown that premalignant adenomas have a low level of proliferation and high level of senescence, while adenocarcinomas are highly proliferative and have low or undetectable levels of senescence [21]. Restoring the activity of tumor suppressors in malignant tissues has been shown to induce a potent senescence response and even tumor regression in some cases. Although the restoration of the activity of tumor suppressors led to partial regression of tumors or even their elimination, the significant expression of these suppressors had a protumorigenic effect [22]. There are reports that the expression of *Ras* and *Wnt5A* in tumors is associated with signs of aging [23], but this information needs to be interpreted correctly. This is just an indication that the mechanisms of self-renewal, initially, being antagonists of the processes of differentiation and aging, through positive feedback, can trigger the processes of differentiation. It is only evidence of the preservation of the way back to differentiation in tumors. It is known that the reversal of tumor cells into normal ones can occur under the influence of the so-called oncogenes [24,25]. This reversion has occurred both under immunoprivileged conditions and in other experiments on the induction of differentiation [26]. Similarly, aging and SASP, known as factors of differentiation and cell cycle arrest, temporarily protect against cancer but trigger the processes of dedifferentiation, pluripotency and rejuvenation at some stage through positive feedback.

Thus, cellular senescence, possibly due to the epigenetic suppression of growth-proliferative genes/signaling pathways and the activation of differentiation genes/cell cycle arrest/apoptosis, temporarily protects against cancer (more correctly, it slows down the cancer incidence). However, SASP signaling and a certain critical level of tissue/function deficiency initiate the reverse process of rejuvenation through recapitulation of the embryonic pathway of development through the epigenetic derepression of the first and suppression of the second pathway (at the molecular level, rejuvenation and pluripotency are the states in which genes responsible for differentiation are suppressed but genes responsible for cell renewal are reactivated). Furthermore, if we can manage to prevent cancer, that is, DG (potency expansion of single structures composing the organism to the detriment of the whole organism), the completion of rejuvenation leads to the disappearance of SASP signaling and returns cells to differentiation through negative feedback from the products of the growth genes. After some time, the SASP-aging signaling then accumulates again, and the process repeats. This closed circle of rejuvenation is the essence of the proposed new paradigm.

## A paradigm shift is due

There are two major problems: the eradication of cancer and aging. For radical rejuvenation, gerontologists attempt to activate signaling pathways for rejuvenation/pluripotency. Quite often, such attempts result in the formation of tumors. This happens because the only way is to radically rejuvenate and this normally, without special intervention, leads to cancer. At the same time, oncologists are trying to suppress all these signaling pathways of rejuvenation, based on the idea that tumor cells are the enemies and that they should be eliminated by all available means. In short, this strategy can be called a killing strategy (both through direct action and creating conditions unfavorable for cell growth and proliferation). This flawed enemy-thinking paradigm is based on misreading the signs that are associated with cancer as the clinical phenomenon of DG itself. This currently applied killing strategy does not restore tissue and function deficiency but rather exacerbates it. That is why, after some clinical success, this strategy leads to a recurrence of cancer and the formation of cell clones that are resistant to therapy [27]. These authors also reported that human mammary carcinoma cells induced to undergo apoptosis could recover with increased tumorigenicity both *in vitro* and *in vivo* and form lymph node metastases. The authors concluded that cancer stem cells arise *de novo* from nonstem cancer cells after apoptosis turnaround, possibly through epigenetic modifications. The recovered cells have undergone epithelial–mesenchymal transitions in the primary tumors *in situ* and mesenchymal–epithelial transitions in the metastatic cells. Chemotherapy-induced senescence could change the stem cell properties of malignant cells, reprogramming nonstem bulk leukemia cells into self-renewing, leukemia-initiating stem cells [28]. In addition, it should be emphasized that chemotherapy (such as with doxorubicin) leads to stromal monocyte chemoattractant protein 1 (CCL2)-induced C chemokine receptor type 2 (CCR2)-mediated attraction of monocytes to tumors [29]. All of these factors provoke tumor relapse. A subpopulation of basal-like human mammary epithelial cells has been identified, which spontaneously dedifferentiates into cancer stem-like cells (CSC-like) [30]. These findings suggest that both cancer nonstem and CSC-like cells can arise *de novo* from differentiated cells, and the hierarchical model of stem cell biology should thus include bidirectional interconversions between stem and nonstem cell states. These results showed that differentiated epithelial cells can return to a stem-like state in mammalian cells. According to the authors, if cancer cells can spontaneously dedifferentiate into CSCs, then targeting CSC populations is unlikely to be successful since the therapeutic eradication of existing CSC populations might be followed by their regeneration from non-CSCs within the tumor after the treatment.

Consequently, what we have in the end as a result of the ‘enemy’-focused paradigm of oncology is neither rejuvenation nor salvation from cancer. This makes sense, since a train cannot move in opposite directions at the same time.

### What then is the solution?

As we noted earlier when presenting our unified model of aging and loss of regenerative potential [2], cancer cells are normal cells with a blocked entry to the normal growth path and redifferentiation, and the last feature is the only marker of malignant growth. It is this blocking and nonlimited execution of a developmental program in reverse order that is the cause of the disintegrative character of its growth or, in other words, the cause that transforms rejuvenation into DG – not the expression of the so-called oncogenes. Oncogene expression does not affect the normal morphogenetic potential of cells. Oncogenes, as genes that cause cancer, do not exist at all. They are normal genes, due to which organisms are developed and due to which they can potentially reach immortality. All properties that are associated with cancer, except blocked redifferentiation, are features of the embryonic pathway recapitulation and self-renewal, and they are inherent for cells at different stages of ontogenesis. In other words, the diversity of the tumor properties is within the range of a normal genome variation that occurs in ontogeny during normal cell development and differentiation. Re-initialization of the program of embryogenesis develops there and then, where and when the current program of development of this organ and tissue is critically insufficient for adequate response and functioning. In other words, it is possible to view cancer as a compensation for this deficiency through the launching of embryonic pathway recapitulation.

Insufficiency of the effective self-renewal and failed adaptive and reparative capability of cells underlie the origin of malignant cell dedifferentiation. The suppression of regenerative cell proliferation is the primary event of initiation of dedifferentiated tissue growth. The transformation of normal cells into tumor cells is an adaptive response to a failure in self-restoration and repair capabilities. Due to the rebirth process, complete tissue renewal leading to the elimination of senescence occurs similarly to embryonic tissue development. We propose to use this potential of transformed cells to eliminate senescence. This will make it possible to direct the process of transformation toward an integrated growth path, to prevent the clinical phenomenon of malignancy and to use the potential of transformed cells to initialize the self-renewal program and program of unlimited life for the whole organism.

### So what shall we propose then?

The first postulate of the new paradigm: The embryonic rejuvenation and DG pathways are essentially the same, but at different stages of ontogenesis, this mechanism is carried out in opposite ways.

As shown in planarians [31], only those neoblasts (Nb2) that express both PIWI-1 and tetraspanin-1 (Tspan-1) have the ability to restore body parts and even the entire body after fragmentation. The authors did not conduct phenotyping of neoblasts, but the fact that the knockdown of Tspan-1 led to the inhibition of neoblast mobilization to amputation sites suggests that the observed effect was due to CD151 and its synergists, CD8, CD9, CD37 and CD63 neoblasts, which play an active role in the neoangiogenesis and metastasis of tumors in carcinogenesis [32–34]. The highly conserved proteins of the Tspan-1 family, found in all Metazoans and re-expressed in an adult body only during carcinogenesis, play an important role in regulating the migration and invasion of cancer cells [35,36]. CD63 plays an active role in the epithelial–mesenchymal transition [37] and suppresses the antigen presentation [38] as CD37, and CD151 suppresses the costimulatory signal during antigen presentation [39]. Importantly, all these conditions are necessary for the tolerance induction to the presented antigens. This provides further evidence of common mechanisms of cancer and rejuvenation. CD9 was absent on NK cells in healthy controls but was upregulated after incubation with TGF-β [40,41]. It should be emphasized that CD9-positive NK cells are usually found in the maternal part of the placenta [42], where they exert immunosuppressive functions. PIWI, like Tspan-1, is re-expressed in CSCs. These cells exhibit epigenetic alterations and signaling pathway profiles that are typical for stem cells, such as self-renewal capacity, rapid proliferation and multilineage differentiation. In this regard, it is believed that *PIWI* acts as a so-called oncogene and is a marker of CSCs due to its restricted expression during embryonic development and aberrant expression in various types of cancer [43]. Re-expression of *PIWI* in cancer is associated with certain already defined CSC-associated proteins and indicates the involvement of all these proteins in the process of tumor growth [44]. A positive correlation between PIWIL1 and OCT4 mRNA levels as well as PIWIL2 and SOX2 levels has been found in colon cancer tissues [45]. At the same time, nonaging tissues exhibit an unlimited ability for self-renewal and genome integrity throughout life, which is mainly due to Piwi–piRNA pathway. This self-renewal pathway was originally discovered in the male *Drosophila* germline, and it resulted in repressing the activity of mobile genetic elements, also called transposable elements (TEs) or ‘jumping’ genes. This pathway is also active in various cell lines, which means that nonaging CSC cells acquire certain germline-specific characteristics. This indicates that soma-to-germline transformation occurs, with the activation of the Piwi–piRNA pathway and an unlimited proliferation capacity. In addition, certain planarians and cnidarians, such as the planarian flatworms and freshwater hydra, express components of the Piwi–piRNA pathway in the soma, leading to the unlimited self-renewal of soma. These organisms can be reproduced clonally, that is, the progeny can actually ‘be regenerated’ from the somatic cells of the parent body. Thus, besides the germline, the Piwi–piRNA pathway is also active in essentially all types of nonaging somatic cells in various organisms, including somatic stem cells of the sponges, jellyfish, planaria (in this organism, totipotent stem cells are called neoblasts), sea slugs, fruit flies (where the germline function of Piwi proteins depends on somatic cells of the gonads), sea squirts and mammals. In humans, the Piwi–piRNA pathway is activated in both cancerous and hematopoietic stem cells. This pathway causes not only TEs silencing but also other various cellular processes, including epigenetic reprogramming, proliferation and self-renewal. It should be emphasized that the transposons responsible for the mutations of the functional DNA segments are effectively suppressed by the Piwi–piRNA mechanism in the nonaging germline and cancer cells. In contrast, aging somatic cells, in which the Piwi–piRNA pathway is inactive, are subject to a high level of transposition and mutations. It should be especially noted that the phylogenetically conservative mechanisms described above unite practically all living organisms, including humans.

Thus, these and other data suggest that the pathways to rejuvenation and cancer are essentially the same. Rejuvenation features and cancer-associated signs are the same (with the exception of blocked redifferentiation), but they do not transform a normal cell into a cancerous one. Trying to kill cancer, we kill rejuvenation. Trying to radically solve the problem of rejuvenation without preventing the blocking of redifferentiation, we inevitably end up with cancer again.

The basis of effective self-renewal is complete, both quantitative and qualitative replacement of aged and lost structures and functions by specific, full-fledged, functionally active structures. The efficiency of regeneration, which is based on definitive tissue-specific induction, weakens with age. Progression of cells toward a more differentiated state and a gradual increase in the level of tissue specialization lead to the narrowing of their adaptive potential spectrum (relative adaptive rigidity), the lowering of the rate of self-renewal, a further deepening in a state of differentiation, the appearance of nonself-reproducing units in self-replicating systems, a reduction of cell and tissue resistance to harmful environmental factors and substantial qualitative changes in the process of reparation (regeneration). As a result, replacement of dead structures with nonspecific, defective structures occurs. The metabolic provision of hyperfunctioning of the remaining structures becomes unsustainable, their functional insufficiency increases, the absolute tissue (organ) deficit reaches a critical level and endogenous intoxication processes are potentiated, making the body even more vulnerable to exogenous factors.

It is impossible to achieve radical rejuvenation without the proper conditions that enable cancer cells to come back to tissue-specific commonality. This is because the increased deficiency of tissue/function and repair processes requires greater flexibility than can be provided within the definitive tissues characterized by firm determination and differentiation. Only embryonic induction, with its full reproduction of the forms and functions of the organs (tissues), will result in complete self-renewal. It becomes clear that the rejuvenation ability is nothing more than the ability for growth; therefore, rejuvenation must be carried out by the embryo-like type of tissue growth.

Considering the above information and the fact that involution processes begin at different times in various organs, are not equally expressed in distinct structures of the same organ and develop at different rates and in different directions, we can answer the question regarding when and why this recapitulation of the embryonic pathway of development occurs. Therein lies the formulation of the second postulate of the new paradigm.

The second postulate of the new paradigm: recapitulation (self-initiation) of the embryonic pathway occurs where and when the program of development of definitive structures cannot result in effective self-renewal. In other words, it starts as a rejuvenation program but is transformed into DG.

Why does this happen? It is due to a blocked way back.

The third postulate of the new paradigm: DG is simply epigenetic-blocked redifferentiation and unfinished somatic embryogenesis/rejuvenation and nothing more.

It has recently been shown [46] that the evolutionarily conserved embryonic program in somatic cells can be derepressed in oncogenesis and the formation of polyploid giant cancer cells, which are typical of many tumors and identical to the preimplantation embryo associated with the activation of senescence. Meanwhile, their daughter cells avoid senescence via time- and space-dependent activation of the expression of the embryonic stem cell markers Oct4, Nanog, Sox2 and SSEA1 through dephosphorylation of a key mediator of the Hippo pathway (YAP), with its subsequent movement into the nucleus. As is well known, *YAP* silencing is related to phosphorylation, inhibition of *YAP* entering the nucleus and decay in the cytoplasm, which inhibits cell division and induces premature cell senescence. It has also been shown that polyploid giant cancer cells are able to differentiate into all three germ layers. This indicates the preservation of the Hippo differentiation signal pathway, the activation/deactivation of which occurs epigenetically [47].

What blocks rejuvenation-like realization of the embryonic pathway and its conversion into DG?

Embryos develop in immunoprivileged sites. Indeed, we believe this is the only fundamental difference that distinguishes the developing embryo, which advances from totipotency to specialization, and the differentiated cells that move in the opposite direction during recapitulation of the embryonic pathway. The data summarized earlier [3] indicate that tumor cells placed in immunoprivileged sites acquire normal differentiated status with a loss of tumorigenicity [48,49] and that the transplantation of the nucleus of a tumor cell (not only embryonic) into the oocyte with subsequent implantation into the immunoprivileged female uterus gives rise to a normal embryo [50–54]. This means that the genetic program in it was not affected or changed. These and other data indicate that the tumor does not develop as a result of immunodeficiency or the immune ‘escape’ phenomenon; on the contrary, it is due to an active immune response to the transformed phenotype of cells, stepped on embryonic self-renewal pathway and the active use of that immune response by those cells for their ‘favorable’ unlimited expansion. In this case, the immune response does not develop according to the canon of classical immunology (when the immune response leads to target cell elimination) but rather according to the active tolerance reaction type or regulatory facilitation reaction [55] (when the immune response leads to the target cell potentiating), such as during regeneration and pregnancy, when the interaction with the immune system leads not to the elimination of target cells but to the activation of growth-proliferative processes. That is, the morphogenetic potential of the immune system is realized, which was almost predicted by Burnet. The morphogenetic function of the immune system is a particular phenomenon of morphostasis. It ensures the maintenance of quantitative tissue homeostasis through the potentiation of proliferation processes during regeneration [56–58]. During regeneration, this potentiation occurs within the limits of tissue-specific differentiation and therefore as an IG (submission of the purpose of the single structures composing the organism to the goal of the whole organism), but in the case of recapitulation of the embryonic pathway and pluripotency, this occurs as a DG.

So how can the immune response convert the rejuvenation process into DG?

As we propose, development only in the immunoprivileged sites ensures the advancement from totipotency to differentiation. IG is carried out through epigenetic activation of genes/signaling differentiation pathways and epigenetic repression of growth-proliferative genes/signaling pathways of pluripotency. It is this fine self-tuning, that is, the stage-specific alternation of on/off of these two opposite signaling pathways through a complex network of positive and negative feedback loops, which results in methylation/demethylation of genes, ensuring the direction of the development vector. This tuning is carried out through the interaction of pluripotency (self-renewal)-related factors (pathways), such as Oct4, Nanog, Sox2, Ronin, Rae28, Meis1, c-Myb, c-myc, Cbp, Gata-2, Mll and Bmi-1, which implement their functions through the blocking of two main pathways of differentiation (the so-called tumor-suppression pathways), p16Ink4 and p19Arf, and factors/pathways of differentiation, such as p53, p21, p27, p15, which implement their function through p16Ink4 and p19Arf (stage-specific on/off alternation of these pathways leads to differentiation and IG, but the separation of self-regulation leads to serious abnormalities in embryogenesis [59]). A similar interaction occurs between the so-called oncogene *BRG1*, which plays an important role in embryonic development (angiogenesis) [60], and p53/p21 [61] as well as between VEGF (angiogenesis) and p53/p21 [62]. Another example of self-tuning, at a certain stage of embryogenesis, is the coordinated inhibition of TGF-β and activation of the cell-cycle inhibitor p21, resulting in the regression and destruction of most parts of the female Wolffian duct, with the significant exception of the caudal portion, which takes part in the formation of the vagina (blocking of this mechanism is associated with infertility); the opposite process occurs in male embryos, leading to the development of the epididymis and vas deferens [63]. It should be noted that p21 is most likely responsible only for arresting the cell cycle in females, as one of the functions of p21 is to prevent apoptosis and p53 is responsible for apoptosis. A similar example of coordinated self-tuning is the LncRNA (antisense LncRNA)-mediated action of polycomb-repressor complexes (PRC1 and PRC2) through selective methylation (e.g., EZH2 subunit of PRC2)/demethylation (e.g., Jarid2 subunit of PRC2). Similar epigenetic regulations have been found for various forms of cancer, such as blocking interactions: ANRASSF1 (RASSF1 antisense RNA)–RASSF1 (tumor-suppressor); ANRIL (antisense noncoding RNA in the INK4 locus)–CDKN2A/B; HOTAIR (HOX transcript antisense RNA)–*HOX* genes; and PTPRG-AS1 (protein tyrosine phosphatase, receptor type, G, antisense)–PTPRG (tumor suppressor). These epigenetic regulations have been found for synergistic interactions: LncRNA and pluripotency factors–SOX2-overlapping transcript (SOX2OT)-mediated expression via PCGF6 (PRC1 complex) of transcription factor SOX2, which is required for maintaining pluripotency. The regulation of the Wnt/β-catenin and PI3K/AKT/mTOR signaling pathways by LncRNA- and NuRD-mediated epigenetic activation of transcription factors/genes *Nanog, Oct4, Sox2, Klf4, Esrrb, Sall4* and *Myc*, supporting pluripotency and the suppression of differentiation genes *Foxl1* and *Dusp4*, was recently described in the literature [64,65]. The role of PIWI–piRNA pathway in the epigenetic regulation of these processes has also been reported [66]. The role of LncRNA in maintaining the pluripotency of embryonic, stem and tumor cells is well known, but it is also known that a number of LncRNAs have the ability to turn on differentiation signaling [67]. For example, prevention of the inhibition of the synthesis of CBX2, CBX4 and CBX8 subunits of the PRC1 is required for differentiation through the repression of CBX7.

Most likely, the dysregulation of these fine self-tuning networks is the cause of the conversion of rejuvenation into DG.

These processes are complex and are not yet fully understood. For example, some authors argue that thymine DNA glycosylase (TDG; a coactivator of *p53* [68]) protects CpG islands from hypermethylation and actively demethylates tissue-specific promoters and enhancers, thereby playing an important role in cancer prevention. According to other authors, TDG interacts with transcription factor TCF4 and acts as a positive regulator in the Wnt pathway, is upregulated in human cancer and is required for cancer growth. These data are consistent with the results, according to which the epigenetic barrier can be overcome by active DNA demethylation through TDG allowing axon regeneration in adult mammals [69]. It is this complex, multifactorial and multivector nature of the interactions described above, without comprehensive knowledge of this network functioning at different stages of development that makes any exogenous intervention in the genome (aimed at correcting it) random, unpredictable and unrepresentative. Only the creation of the conditions for all of this to happen – both fine self-tuned and self-regulated (as in embryogenesis) – can result in the radical solution of cancer treatment and real antiaging.

The fourth (and main) postulate of the new paradigm: The immune response (by type of active tolerance) to the markers of the embryonic developmental pathway causes an epigenetic blockage of the redifferentiation potential and the conversion of the rejuvenation pathway into the DG (cancer). Therefore, the induction of complete immunological tolerance to these markers (the so-called tumor-associated antigens) will make it possible to escape this conversion.

## Conclusion

To prevent the transformation of the rejuvenation path into the DG, it is necessary to create conditions that mimic the immunoprivileged conditions of fetal development through the induction of immunological tolerance to antigens associated with embryonic rejuvenation. An agent that can simultaneously turn on the rejuvenation process and can block DG (cancer) must have several features, in particular, tolerogenic properties, rejuvenation turn-on signal capabilities, the ability to start the process of removing by-products associated with the SASP from cells and the extracellular matrix and the ability to protect transformed cells from the host immune response.

## Future perspective

We believe that only this method of aging elimination has real prospects for a future that is fraught with cancer, as we will be able to convert this risk into rejuvenation through the continuous cycling of cell dedifferentiation–differentiation processes, under the following conditions:

- Without direct intervention in the genome/epigenome
- Through recapitulation of morphogenetic modules of early stages of ontogenesis
- Through prevention of malignant conversion of this recapitulation and directing it into rejuvenation (completed somatic embryogenesis)

At the cellular level, this will eliminate senescence, as the final stage of cells’ fate, making it only a transitional stage. At the level of the whole body, it will eliminate aging.

This is real, since ‘crucial regeneration-driving genes are present in most animal genomes, implying the existence of an innate ability to regenerate lost tissues in most animals. Thus, a key aspect of diverse regenerative capacities is not the presence or absence of regeneration genes, but in the mechanisms controlling the activation of these genes after injury’ [70].

The currently proposed approach is being rigorously tested in animal experiments.

Further justification of the concept will be presented in part two of our publication with the subtitle, ‘Rejuvenation Circle.’

### Definitions

IG – Integrating Growth, is defined here as the submission of potency of single cells composing an organism to the development program and functions of the whole organism.

DG – Disintegrating Growth, is defined here as a priority of extension potency of single cells over the development program and functions of the whole organism.

**Executive summary**

**Four postulates of the new paradigm:**

- The embryonic rejuvenation and disintegrating growth (DG) pathways are essentially the same, but at different stages of ontogenesis, this mechanism is carried out in opposite ways.
- Recapitulation (self-initiation) of the embryonic pathway occurs where and when the program of development of definitive structures cannot result in effective self-renewal. In other words, it starts as a rejuvenation program but is transformed into DG.
- DG is simply epigenetic-blocked redifferentiation and unfinished somatic embryogenesis/rejuvenation and nothing more.
- The immune response (by type of active tolerance) to the markers of the embryonic developmental pathway causes an epigenetic blockage of the redifferentiation potential and the conversion of the rejuvenation pathway into the DG (cancer).
